# Genomic Characterization of a Novel SARS-CoV-2 Lineage from Rio de Janeiro, Brazil

**DOI:** 10.1128/JVI.00119-21

**Published:** 2021-04-26

**Authors:** Carolina M. Voloch, Ronaldo da Silva Francisco, Luiz G. P. de Almeida, Cynthia C. Cardoso, Otavio J. Brustolini, Alexandra L. Gerber, Ana Paula de C. Guimarães, Diana Mariani, Raissa Mirella da Costa, Orlando C. Ferreira, Covid19-UFRJ Workgroup, LNCC Workgroup, Adriana Cony Cavalcanti, Thiago Silva Frauches, Claudia Maria Braga de Mello, Isabela de Carvalho Leitão, Rafael Mello Galliez, Débora Souza Faffe, Terezinha M. P. P. Castiñeiras, Amilcar Tanuri, Ana Tereza R. de Vasconcelos

**Affiliations:** a Departamento de Genética, Instituto de Biologia, Universidade Federal do Rio de Janeiro, Rio de Janeiro, Brazil; b Laboratório de Bioinformática, Laboratório Nacional de Computação Científica, Petrópolis, Brazil; c Laboratório Central de Saúde Pública Noel Nutels, Rio de Janeiro, Brazil; d Secretaria Municipal de Saúde de Maricá, Maricá, Brazil; e Secretaria Estadual de Saúde do Rio de Janeiro, Rio de Janeiro, Brazil; f Faculdade de Medicina, Universidade Federal do Rio de Janeiro, Rio de Janeiro, Brazil; g Instituto de Biofísica, Universidade Federal do Rio de Janeiro, Rio de Janeiro, Brazil; Cornell University

**Keywords:** P.2, E484K, genomic surveillance, COVID-19

## LETTER

Almost simultaneously, several studies reported the emergence of novel severe acute respiratory syndrome coronavirus 2 (SARS-CoV-2) lineages characterized by distinct phylogenetic and genetic features (1–4). Here, we sequenced 180 complete genomes collected between April and November 2020 from the Brazilian state of Rio de Janeiro (see Table S1 in the supplemental material) and identified 37 samples composing a new variant lineage. This novel lineage, currently classified as P.2 by Pangolin (COG-UK), a descendant of the B.1.1.28 strain, is distinguished by five lineage-defining mutations: 5′ untranslated region [UTR] C100U, (ORF1ab L3468V, ORF1ab synC11824U, S E484K, and N A119S). Five other mutations were also detected in many genomes from this lineage (ORF1ab L3930F, ORF1ab synA12964G, ORF8 synC28253U, N M234I, and 3′ UTR C29754U).

We found a total of 731 single-nucleotide variants (SNVs) across the 180 samples, of which 50.3% were missense, 44.5% synonymous, 5.1% intergenic, and 0.1% nonsense. For the phylogenetic reconstruction, we gathered a global data set containing the 180 new genomes plus 1,197 high-coverage genomes from GISAID (GISAID Initiative; see Table S2), including 116 Brazilian and 1,081 worldwide genomes whose collection dates ranged from May to November (data downloaded 3 December 2020). Genomes were classified using Pangolin (COG-UK).

**FIG 1:**
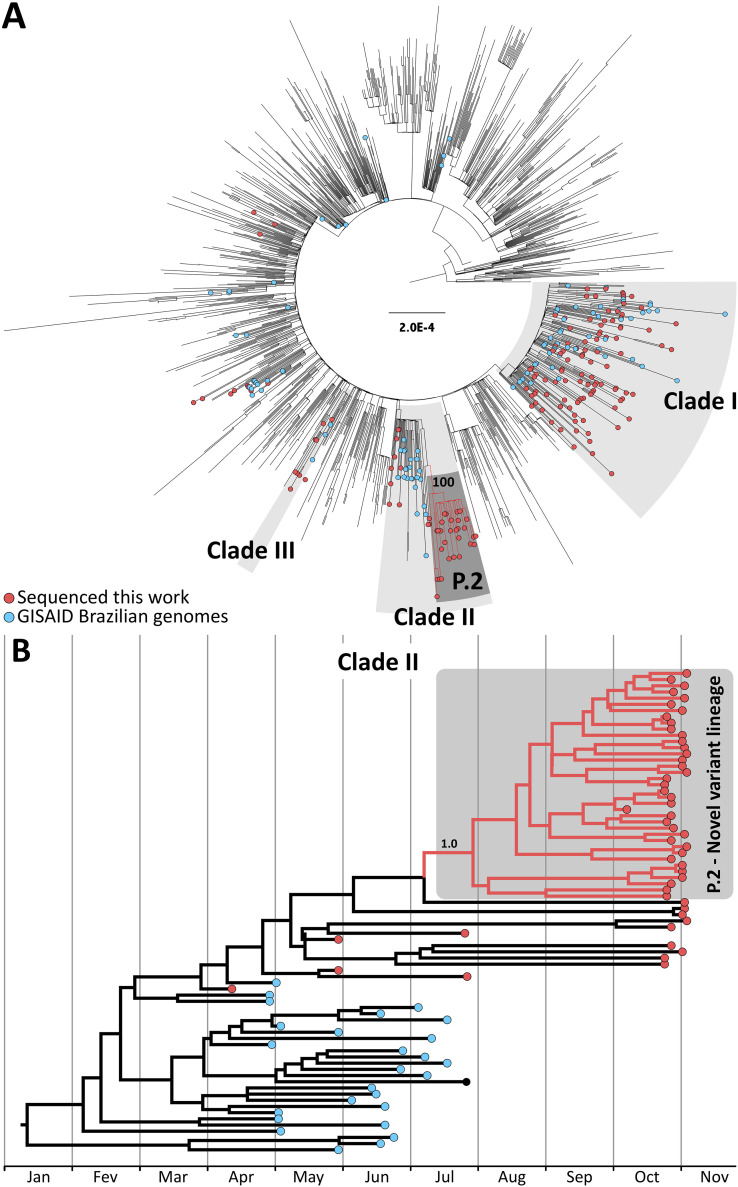
Phylogeny and timescale of P.2 lineage. (A) Maximum-likelihood tree of 1,377 SARS-CoV-2 genomes, including the 180 new Brazilian genomes generated in this work. We randomly sampled one genome/week/country from available samples in GISAID with collection dates ranging from May to November. Brazilian sequences with collection dates from May to November were all added to the data set (see Table S2). We ran IQTree 2.0.3 (10) under a general time-reversible (GTR) model of nucleotide substitution (11) with empirical base frequencies and invariant sites. Tips are colored according to their origin. Genomes generated from this work are red, other Brazilian genomes are blue, and genomes from other countries are not colored. Gray areas represent the three clades where Brazilian viruses are concentrated. The emergent lineage identified in this work, P.2, is in a dark-gray box and highlighted with red branches. The approximate likelihood ratio test (aLRT) support value for the branch holding P.2 monophyly is shown. (B) A time-scaled tree of clade II was estimated under a strict molecular clock in BEAST v.1.10.4 (12) using the GTR+I (11) nucleotide substitution model and assuming an exponential growth tree prior and a normal prior for the clock rate (mean = 8 × 10^−4^ and standard deviation = 0.1 × 10^−5^). The convergence of the Markov chain Monte Carlo chains, which were run at least for 50 million generations and sampled every 1,000th step, was inspected using Tracer v.1.7.1 (13). Maximum clade credibility (MCC) summary trees were generated using TreeAnnotator v.1.10.4 (12). Red circles in the tip nodes represent genomes generated in this work, and blue circles indicate other Brazilian samples. P.2 is identified by the red branches and highlighted in a gray box. The posterior probability of the branch holding P.2 monophyly is shown.

The phylogenetic reconstruction indicates that the vast majority (86.8%) of Brazilian genomes fall within three clades (Fig. 1). Clade I comprises B.1.1.33 strains, while clades II and III are composed of B.1.1.28 strains. We inferred that P.2 falls within clade II (Fig. 1) and emerged in July 2020.

**FIG 2:**
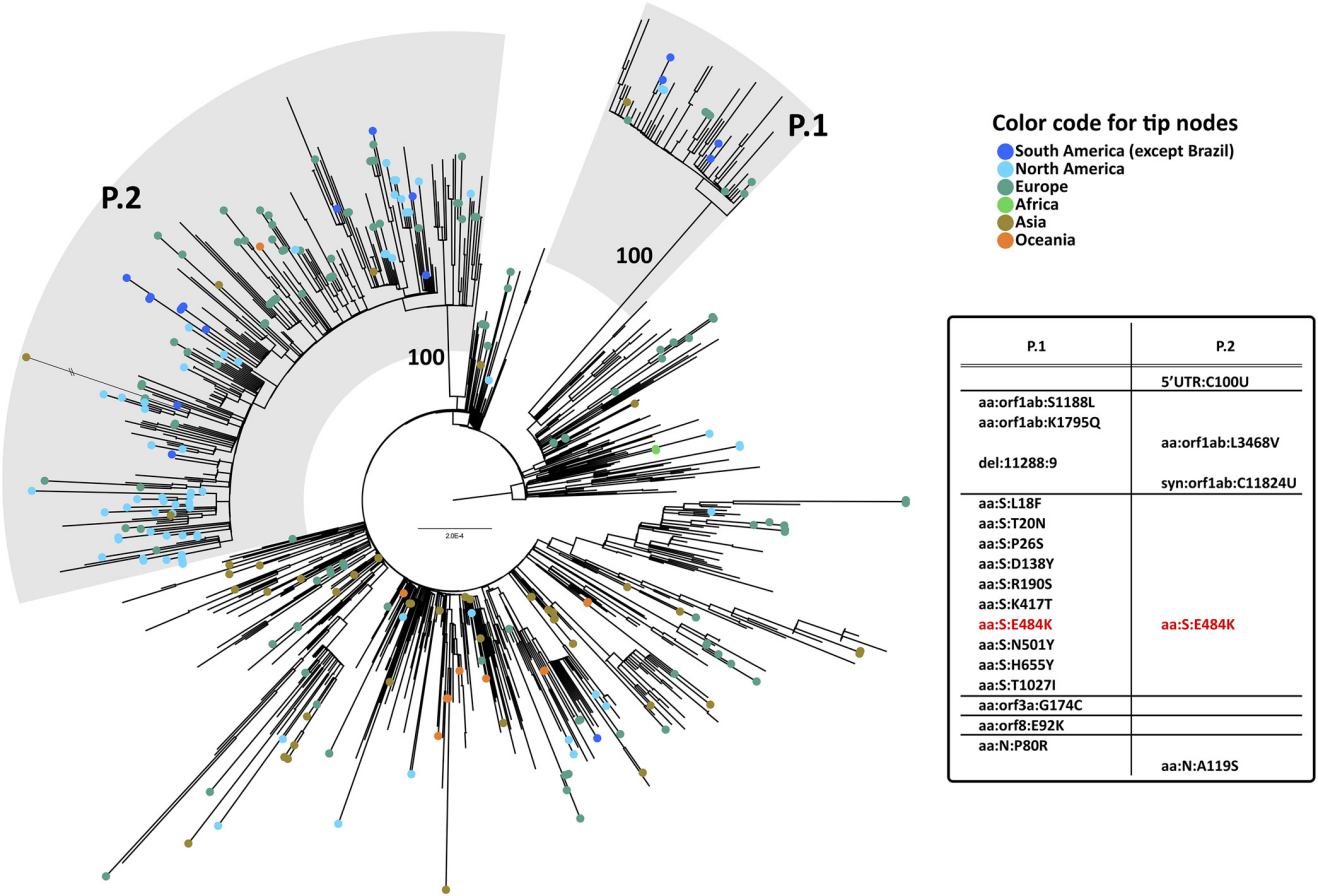
Phylogeny of B.1.1.28 and its alias lineages (P.1 and P.2). Maximum-likelihood tree of 993 SARS-CoV-2 genomes. We downloaded all B.1.1.28, P.1, and P.2 high-coverage genomes available in GISAID on 11 February 2021. The phylogeny was estimated using IQTree 2.0.3 (10) under a GTR+F+R3 model chosen by ModelFinder. Brazilian genomes are not tip colored. Viruses from other countries are colored according to their geographical origin (dark blue, South America; light blue, North America; dark green, Europe; light green, Africa; brown, Asia; and orange, Oceania). Gray areas highlight P.1 and P.2 lineages. The aLRT support value for the branch holding P.1 and P.2 monophyly is shown. The tree is rooted with 3 B.1.1 genomes. The chart shows the single nucleotide polymorphisms (SNPs) that characterize P.1 and P.2 lineages.

An essential feature of the new strains recently described in the United Kingdom (B.1.1.7) (1), South Africa (B.1.351) (2), and Brazil (P.1) (3, 4) is that they have a unique set of multiple spike mutations, including N501Y, which is associated with greater infectivity and transmissibility. In contrast, all P.2 genomes exhibit only E484K in addition to a varying collection of either novel or rarely found spike mutations. E484K is also present in P.1 and B.1.351 lineages, but not in B.1.1.7. Since both P.1 and P.2 lineages are descendants of B.1.1.28 and both were first detected in Brazil, we performed a phylogenetic analysis of all their genomes available in GISAID (data downloaded 11 February 2021). Figure 2 highlights the differences between them and their origin, showing that despite sharing E484K, both emerged from independent events. The localities where these two lineages were first detected have had entirely different epidemiological dynamics since the beginning of coronavirus disease 2019 (COVID-19) history in Brazil (5); therefore, the two variants arose independently in different epidemiological contexts.

The cases of reinfection involving the new variant lineage P.2 (6, 7) and the ability of viruses harboring the mutation E484K to escape from neutralizing antibodies (8, 9) emphasize the importance of monitoring the spread of this new strain. In about 4 months, 342 genomes classified as P.2 have become available. The emergent lineage P.2, initially detected in 37 samples from Rio de Janeiro State, is spread across all Brazilian regions and 16 other countries. Brazilian viruses are the oldest and constitute the majority (55%) of the P.2 genomes, suggesting its exportation worldwide.

The present study was approved by the National Committee of Research Ethics and by the Ethics Committee of Hospital Universitário Clementino Fraga Filho (protocol numbers 30161620.0.1001.5257 and 34025020.0.0000.5257).

## 

### Data availability.

NGS data generated in our study are publicly available in SRA-NCBI (www.ncbi.nlm.nih.gov/sra), under BioProject accession no. PRJNA686081. Genome sequences were also deposited in GISAID (https://www.gisaid.org/) and are fully accessible for registered users within the “browse” option of the EpiCoV database (see Table S1).

## Supplementary Material

Supplemental file 1

## References

[1] Rambaut A, Loman N, Pybus O, Barclay W, Barrett J, Carabelli A, Connor T, Peacock T, Robertson DL, Volz E, on behalf of COVID-19 Genomics Consortium UK. 2020. Preliminary genomic characterization of an emergent SARS-CoV-2 lineage in the UK defined by a novel set of spike mutations. https://virological.org/t/preliminary-genomic-characterisation-of-an-emergent-sars-cov-2-lineage-in-the-uk-defined-by-a-novel-set-of-spike-mutations/563.

[2] Tegally H, Wilkinson E, Giovanetti M, Iranzadeh A, Fonseca V, Giandhari J, Doolabh D, Pillay S, San EJ, Msomi N, Mlisana K, von Gottberg A, Walaza S, Allam M, Ismail A, Mohale T, Glass Aj Engelbrecht S, Van Zyl G, Preiser W, Petruccione F, Sigal A, Hardie D, Marais G, Hsiao M, Korsman S, Davies M-A, Tyers L, Mudau I, York D, Maslo C, Goedhals D, Abrahams S, Laguda-Akingba O, Alisoltani-Dehkordi A, Godzik A, Wibmer CK, Sewell BT, Lourenço J, Alcantara LCJ, Kosakovsky PS, Weaver S, Martin D, Lessells RJ, Bhiman JN, Williamson C, de Oliveira T. 2020. Emergence and rapid spread of a new severe acute respiratory syndrome-related coronavirus 2 (SARS-CoV-2) lineage with multiple spike mutations in South Africa. medRxiv 2020.12.21.20248640.

[3] Faria NR, Claro IM, Candido D, Franco LAM, Andrade PS, Coletti TM, Silva CAM, Sales FC, Manuli ER, Aguiar RS, Gaburo N, Camilo CC, Fraiji NA, Crispim MAE, Carvalho MPSS, Rambaut A, Loman N, Pybus OG, Sabino EC, on behalf of CADDE Genomic Network. 2021. Genomic characterisation of an emergent SARS-CoV-2 lineage in Manaus: preliminary findings. https://virological.org/t/genomic-characterisation-of-an-emergent-sars-cov-2-lineage-in-manaus-preliminary-findings/586.

[4] Naveca F, Nascimento V, Souza V, Corado A, Nascimento F, Silva G, Costa A, Duarte D, Pessoa K, Gonçalves L, Brandão MJ, Jesus M, Fernandes C, Pinto R, Silva M, Mattos T, Wallau GL, Siqueira MM, Resende PC, Delatorre E, Gräf T, Bello G. 2021. Phylogenetic relationship of SARS-CoV-2 sequences from Amazonas with emerging Brazilian variants harboring mutations E484K and N501Y in the Spike protein. https://virological.org/t/phylogenetic-relationship-of-sars-cov-2-sequences-from-amazonas-with-emerging-brazilian-variants-harboring-mutations-e484k-and-n501y-in-the-spike-protein/585.

[5] Buss LF, Prete CA, Jr, Abrahim CMM, Mendrone A, Jr, Salomon T, de Almeida-Neto C, França RFO, Belotti MC, Carvalho MPSS, Costa AG, Crispim MAE, Ferreira SC, Fraiji NA, Gurzenda S, Whittaker C, Kamaura LT, Takecian PL, da Silva Peixoto P, Oikawa MK, Nishiya AS, Rocha V, Salles NA, de Souza Santos AA, da Silva MA, Custer B, Parag KV, Barral-Netto M, Kraemer MUG, Pereira RHM, Pybus OG, Busch MP, Castro MC, Dye C, Nascimento VH, Faria NR, Sabino EC. 2021. Three-quarters attack rate of SARS-CoV-2 in the Brazilian Amazon during a largely unmitigated epidemic. Science 371:288–292. 10.1126/science.abe9728. 33293339 PMC7857406

[6] Nonaka CKV, Franco MM, Gräf T, Mendes AVA, de Aguiar RS, Giovanetti M, de Freitas Souza BS. 2021. Genomic evidence of a Sars-Cov-2 reinfection case with E484K spike mutation in Brazil. Emerg Infect Dis 10.3201/eid2705.210191. PMC8084516 33605869

[7] Resende PC, Bezerra JF, Vasconcelos RHT, Arantes I, Appolinario L, Mendonça AC, Paixao AC, Rodrigues ACD, Silva T, Rocha AS, Pauvolid-Corrêa A, Motta FC, Teixeira DLF, Carneiro TFO, Freire Neto FP, Herbster ID, Leite AB, Riediger IN, Debur MC, Naveca FG, Almeida W, Livorati M, Bello G, Siqueira MM. 2021. Spike E484K mutation in the first SARS-CoV-2 reinfection case confirmed in Brazil, 2020. https://virological.org/t/spike-e484k-mutation-in-the-first-sars-cov-2-reinfection-case-confirmed-in-brazil-2020/584.

[8] Greaney AJ, Loes AN, Crawford KHD, Starr TN, Malone KD, Chu HY, Bloom JD. 2021. Comprehensive mapping of mutations to the SARS-CoV-2 receptor-binding domain that affect recognition by polyclonal human serum antibodies. Cell Host Microbe 10.1016/j.chom.2021.02.003. PMC7869748 33592168

[9] Wibmer CK, Ayres F, Hermanus T, Madzivhandila M, Kgagudi P, Lambson BE, Vermeulen M, van den Berg K, Rossouw T, Boswell M, Ueckermann V, Meiring S, von Gottberg A, Cohen C, Morris L, Bhiman JN, Moore PL. 2021. SARS-CoV-2 501Y.V2 escapes neutralization by South African COVID-19 donor plasma. bioRxiv 10.1101/2021.01.18.427166. 33654292

[10] Minh BQ, Schmidt HA, Chernomor O, Schrempf D, Woodhams MD, von Haeseler A, Lanfear R. 2020. IQ-TREE 2: new models and efficient methods for phylogenetic inference in the genomic era. Mol Biol Evol 37:1530–1534. 10.1093/molbev/msaa015. 32011700 PMC7182206

[11] Tavaré S. 1986. Some probabilistic and statistical problems in the analysis of DNA sequences. Lect Math Life Sci 17:57–86.

[12] Suchard MA, Lemey P, Baele G, Ayres DL, Drummond AJ, Rambaut A. 2018. Bayesian phylogenetic and phylodynamic data integration using BEAST 1.10. Virus Evol 4:vey016. 10.1093/ve/vey016. 29942656 PMC6007674

[13] Rambaut A, Drummond AJ, Xie D, Baele G, Suchard MA. 2018. Posterior summarization in Bayesian phylogenetics using Tracer 1.7. Syst Biol 67:901–904. 10.1093/sysbio/syy032. 29718447 PMC6101584

